# Selective Change Driven Imaging: A Biomimetic Visual Sensing Strategy

**DOI:** 10.3390/s111111000

**Published:** 2011-11-22

**Authors:** Jose A. Boluda, Pedro Zuccarello, Fernando Pardo, Francisco Vegara

**Affiliations:** Departament d’Informàtica, Escola Tècnica Superior d’Enginyeria, Universitat de València, Avd. Vicente Andres Estellés, s/n, 46100 Burjassot, València, Spain; E-Mails: Jose.A.Boluda@uv.es (J.A.B.); Pedro.Zuccarello@uv.es (P.Z.); Francisco.Vegara@uv.es (F.V.)

**Keywords:** CMOS image sensor, event-based vision, biomimetics, motion analysis

## Abstract

Selective Change Driven (SCD) Vision is a biologically inspired strategy for acquiring, transmitting and processing images that significantly speeds up image sensing. SCD vision is based on a new CMOS image sensor which delivers, ordered by the absolute magnitude of its change, the pixels that have changed after the last time they were read out. Moreover, the traditional full frame processing hardware and programming methodology has to be changed, as a part of this biomimetic approach, to a new processing paradigm based on pixel processing in a data flow manner, instead of full frame image processing.

## 1. Introduction

Nature is a source of inspiration for engineering in general, and particularly in the sensor design field [1]. Evolution, during billions of years, has developed and optimized incredibly efficient sensing systems. Imitation of living things is a smart way to take advantage of the process of natural selection that has made living things the most perfect machines that no engineer is capable of developing. The term biomimetic, coined by Schmitt [2], is generally used as the study and imitation of natures methods, mechanisms, and processes.

The basic principles of how biological systems relate to their environment can be assumed as a working model to design different kinds of sensor technologies. Nevertheless, not all biomimetic sensors work exactly according to the biological principles that are being mimicked. Engineers can decide, when the biological system is understood, whether it would be better to mimic the overall functioning of the biological system, or, to mimic the global strategy of the living things following a different implementation.

Currently, most commercial optical sensors do not follow any biological design guideline. The efforts of engineers and scientists have been mainly focused on achieving high-resolution Cartesian sensors or ultra high-speed cameras [3–6]. In this way, optical sensors designed by humans have evolved from the concept of single frame photograph towards a sequence of snapshots such as a video sequence. It seems that the concept of still frame or snapshot at instant *t* does not exist in most biological systems in the initial stages of the visual process. Moreover, the sequential pixel acquisition and transmission, inherited from the CCD sensor structure [7], is very far removed from the real functioning of a living beings visual system, based on the individual response of the sensing elements [8,9].

Conventional cameras, together with their image processing systems, are commonly used as the sensing stage in many applications. All the images are acquired and transmitted sequentially, whether or not there have been image changes. Moreover, all the computing operations are applied in every case, even if no changes were observed; this being a waste of time and resources.

As an example, let us consider the motion analysis problem from the image processing point of view. From amongst the different approaches for addressing this problem, we can consider the most relevant to be: differential methods, frequency domain schemes, and extraction and matching of relevant points [10]. The normal procedure implies the application of several preprocessing filters on the entire image and for each image in the sequence. Afterwards, more complex processing stages are applied. Whether there have been a lot of changes in the image, only a few, or even no changes at all, the sequence of instructions is systematically applied to the entire image.

The Selective Change-Driven (SCD) approach mimics the data reduction that follows the visual systems of living beings by avoiding the transmission of complete frames [11]. Instead, the pixels’ address and current illumination level are transmitted in descending order as per their illumination level difference with respect to the last transmitted value. Therefore, bandwidth and processing resources can be adapted in such a way that the most significant changes in the environment will be processed, discarding minor variations. We are mimicking the final goal of selective reduction of information but with a trade-off with current technology. The objective has been to take advantage of the capabilities of the technology, looking at biological systems for inspiration, but not as an objective.

A SCD system is mainly, but not only, made up of a SCD camera. Also the processing system is essentially different from a conventional one. A normal camera delivers a sequence of images, whilst a SCD camera delivers a flow of pixels. In this way, it is also possible to implement a data flow policy in the algorithm execution, processing only those pixels that have changed. A pixel only triggers the instructions that depend on it. This strategy will decrease the total amount of instructions to be performed, thus speeding up the algorithm execution.

The remainder of this paper is organized as follows. In Section 2, we give a review of other biomimetic visual sensors. Section 3 presents the SCD sensor, the overall system architecture and some SCD algorithm design considerations. Section 4 shows several experimental results with a simple motion detection algorithm. Finally, Section 5 discusses the conclusions.

## 2. Previous Biologically Inspired Visual Sensors

There have been many bio-inspired optical sensors since the late 1980s. For example, space variant visual sensors are biologically inspired sensors that present a selective reduction of information. The sensor layout has more sensing elements in the center and fewer in the periphery, the log-polar transformation being directly implemented through a sensor which has the log-polar sensor distribution. These kinds of sensors present, in some cases, interesting mathematical properties [12–15]. Many times, this data reduction strategy is related to active vision, another biomimetic approach. An active vision system is able to manipulate its viewpoint in order to focus on the area of interest [16].

On the other hand, one of the characteristics of the visual system of living beings is that the sensing part also incorporates some pre-processing tasks [8]. This smart processing included in the sensing part has been imitated by constructing artificial retinas that incorporate some processes into the sensor surface [17], or even incorporating post-processing stages to implement smart sensors based on hybrid analog-digital architectures [18]. These sensors are mostly built in CMOS technology to implement this on-chip processing capability [19].

Following these ideas of adding some preprocessing task in the sensor plane, the neuromorphic approach appeared. Neuromorphic chips can be described as compact efficient electronic circuits which follow some of the neural systems working principles. A neuromorphic chip copies, or *morphes* as the related scientific community states, the structure of neural connections into silicon circuits [20]. The goal of these kinds of optical sensors is to implement the characteristics of biological vision systems into electronic hardware. To do this, Neuromorphic chips process images directly at the focal plane level with circuits that implement hardware models of the first stages of visual processing in biological systems. These chips have a physical organization similar to the retina: photoreceptors, memory elements, and processing elements share the same physical space on the silicon surface being combined into local circuits that process, in real-time, different types of spatio-temporal computations on the continuous analog brightness signal [21]. Neuromorphic sensors reduce the computations needed for visual perception by extracting only information relevant to the post-processing stages. Additionally, mechanisms for improving electronic imaging are also obtained by mimicking their biological equivalents [22].

The implementation of neuromorphic systems would not be possible without the Address-Event Representation (AER) asynchronous communication protocol [23–25]. This protocol is used for implementing the large amount of connections between huge populations of neurons in different areas. The basic idea of AER applied to visual sensors is that every time a particular photocell has some information to transmit, a transmission request is triggered. In this way the address of the cell is transmitted onto a common serial bus, once this request is granted. Each photocell is coded with a particular address that identifies itself to the cell-matrix. Typically, event-information is coded through the rate-coding scheme. With this method, the density of spikes per unit of time produced by a photocell is proportional to the event to be transmitted (intensity variation, *etc.*) [26].

In the last two decades, many neuromorphic chips have been developed with the idea of performing some spatio-temporal on-plane processing, replacing frame acquisition with individual pixel delivery [27–33]. Some of them have mimicked the selective attention mechanism followed by the active vision principles [34–36]. Others have been used successfully for some biomimetical applications [37–39]. Redi *et al*. [40] presented a sensor where pixels are delivered in order according to the change in the magnitude of the contrast gradient, therefore focusing on the reduction of spatial redundant information. In that work, pixels are read-out asynchronously.

In recent years, a lot of progress has been made in developing complete neuromorphic systems, which mimic not only the sensing part, but also the processing part [41,42]. Of note are the results of the CAVIAR project [43] that developed a general AER infrastructure for constructing bio-inspired hierarchically structured multi-chip systems for sensing, processing and acting. The implementation of the Dynamic Vision Sensor is one of the contributions of this project [27].

The future could be even more promising, combining neural networks with the neuromorphic approach. Convolutional Networks (ConvNets) [44] can be implemented in hardware using spiking neural network techniques and AER technology for pattern and object recognition tasks with minimal latency [45]. For example in [46], it is proposed the first bio-inspired six layer ConvNet that can be implemented with spike-based electronic devices which are already physically available.

The read-out and processing strategy described in this paper is also inspired by the basic principles of the biological visual system: only the address and grey level of changing visual information considered as relevant is selected for transmission and processing. The main difference with other previous works in this field, specifically with the neuromorphic approach, is that the information is ordered previous to transmission, and that pixels are synchronously delivered out of the sensor. One of the key points of this strategy is that the temporal contrast value used to sort the pixel flow is not calculated instantly. That is, the contrast is not the change in illumination between one frame and the next one. The contrast for a particular pixel is represented as the change in illumination between the present frame and the last read-out value for that pixel. This means that the lighting information of a certain pixel will not be lost, and that all events will be processed sooner or later. Synchronous read-out guaranties a continuous pixel flow compatible with most of the current standard available hardware for storage and processing.

## 3. Selective Change-Driven System

A SCD system needs, as its central part, a sensor that implements the pixel stream delivery, ordered by its change magnitude. This visual behavior can be implemented in several ways. The most obvious is by simulating its behavior using software with a high-speed conventional camera and extra storage space at the processing system. Each frame is compared with the previous one, and the pixels that have changed are delivered (grey level and coordinates) ordered by the magnitude of their change. This solution, useful for algorithm evaluation purposes, was adopted initially in this project [47–49]. Unfortunately, the required time for implementing the SCD behavior is a disadvantage that compensates for the speed-up achieved with the data flow algorithm.

### 3.1. SCD Sensor

**Figure 1.**
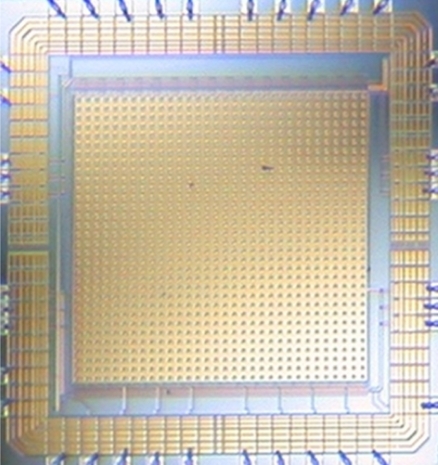
SCD vision sensor layout.

The only solution to obtain all the advantages that SCD offers is to make a custom integrated circuit sensor that follows the SCD acquiring-delivering policy. A new visual sensor which implements this biologically inspired behavior has been designed and fabricated using austriamicrosystems 0.35 *μ*m in CMOS technology. Figure 1 shows the sensor die which has an area of 7.87 mm^2^ (2.8 mm × 2.8 mm). The sensor has a resolution of 32×32 pixels and, although this resolution can be considered low for most applications, it is adequate for demonstration purposes and may even be useful in several cases, such as in resource-limited systems [50]. As shown in Figure 1, most of the sensor layout is hidden, since the pixel circuitry is covered by a metal layer to protect it from the light. There is only a small window to allow the light to reach the photodiode which is the only exposed element in the sensor.

**Figure 2.**
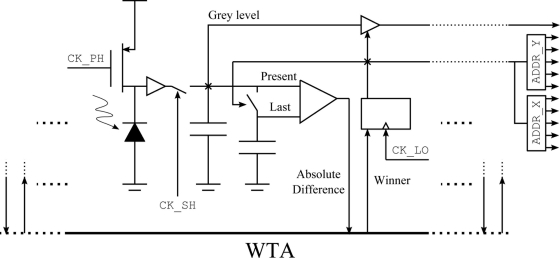
Single pixel schema.

Figure 2 shows a single pixel schema, which has the following parallel functioning: The signal CK PH initiates the charge of a capacitor to a fixed voltage. Afterwards, this capacitor discharges during an integration time (500 *μ*s to 2,000 *μ*s) through the photodiode. After this integration time, the voltage drop in the capacitor is proportional to the light intensity. This voltage is stored in another capacitor using the signal CK SH to activate the sample and hold circuit. The data acquired can be delivered at the same time that a new acquisition is being performed. Then, the captured value (Present value at Figure 2) is compared (subtracted and afterwards rectified) with the last read-out value.

The absolute differences of the 1,024 pixels are compared in parallel using a Winner Takes All (WTA) circuit. The WTA circuit has 1,024 inputs with all the pixels’ absolute differences, and also has 1,024 outputs or winner signals that pinpoint which pixel has won the competition. This circuit selects in a 10 *μ*s a single winner with the greatest change in illumination [51].

After a 10 *μ*s cycle the competition finishes activating a single winner signal. This signal is latched by the CK LOGIC clock. The winner-latched signal updates the capacitor of the winning pixel, storing the last read-out value. Additionally, this signal enables the analog grey level output and activates the *x* and *y* position addresses, which are coded in two digital 5-bit buses. All the sensor control signals are generated with a 32-bit PIC microcontroller running at 80 MHz, which is connected to a computer through an USB link. The grey level is converted to a digital value using 10-bit analog to digital converter included in the microcontroller. The pixel read-out time is around 10 *μ*s, the WTA functioning time, which gives a pixel rate of 100,000 pixels per second.

As has been introduced in Section 2, there are sensors that share the same final goal, the neuromorphic approach being the most important. In our opinion, there are several relevant differences between preceding developments and the SCD policy that must be pointed out explicitly. Every pixel has a memory of the last read-out value, in this way, the choice of the most interesting pixel is based on global change over time instead of just transient delivering. As pixels are ordered previous to read-out, and previously delivered values are stored in memory, the bandwidth for transmission and processing can be adjusted without any substantial loss of information.

Another key difference, compared to previous sensors, is that images are acquired synchronously. The events are then synchronously delivered, ordered by the absolute magnitude of their change, hence presenting a very simple digital interface. This sensor strategy allows the pixel delivery rate to be adjusted to the systems computational capabilities. Moreover, this way of functioning allows for non-accurate functioning; the most important pixel changes of each frame are processed and the minor changes are discarded. This way of functioning could be acceptable, in some cases, if there is no other way to compute the results.

### 3.2. SCD Vision Algorithm Design Considerations

Designing image processing algorithms using the SCD formalism requires a change in the way of thinking about how the programming instructions are applied to data. The SCD methodology can be initially applied to any image processing algorithm, but being realistic, the kind of algorithms that most benefit from this biomimetic redundant data reduction are those that have changes in the image as a main parameter. In this way, it can be considered that the sensor presented in this paper takes advantage of the fact that illumination variations drive movement detection.

A standard motion analysis algorithm can be modeled as a pipeline of successive different transformations to the image flow. First stages are typically some preprocessing tasks such as image smoothing or enhancement. Subsequently, there are some stages that extract some features such as borders, edges, *etc.* Following this there are stages that, in some cases, try to extract movement information, differentiating these features or making a correspondence between relevant points in different frames. Between these processing stages intermediate values are stored. Most of them can be understood as intermediate images, as smoothed images or edge images, but others can be more complex data which is not viewable in a straightforward way. In these cases we call all the intermediate results intermediate images. Therefore, each stage of the image processing pipeline has, as input, full intermediate images, and also produces full intermediate images as output, except the first one which has as input the initial video flow. In a classical approach no matter whether or not the initial pixels or intermediates results have changed, all the instructions at each stage are unavoidably applied to the data, even if they do not generate any change.

**Figure 3.**
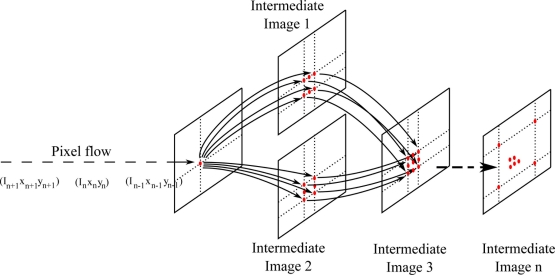
SCD implementation of a generic image processing algorithm.

The classical way of programming image processing algorithms is related to the model of imperative programming, followed by almost all the image processing algorithms, that describes computations in terms of instructions that change a program state. All conventional computer hardware is designed to execute machine code written in an imperative form. This manner of functioning is close to mathematical abstractions but very far from any natural system. Living things are nearer to a reactive or data flow system than a von Neumann machine that implements the imperative paradigm of programming. The SCD execution flow is related to data flow architectures, or in other words, each new pixel fires all the instructions related with this new data. If there are no data changes no instructions are fired. Initially the SCD camera delivers those pixels that have changed (grey level and coordinates). Then the first stage updates the contribution of this new pixel value to its output intermediate images. Following this idea all the stages do the same. When new input data arrives at any intermediate stage, all the related instructions are fired, updating the output intermediate images. Figure 3 shows the data flow in a SCD algorithm. Initially, there is a pixel-flow instead of a full-frame data flow. Each pixel triggers the instructions related to its value, updating the dependent intermediate image. A SCD image algorithm is implemented as a pipeline of intermediate images or feature images, the last image being the algorithm result.

There have been other previous implementations of the data flow paradigm, or event-based functioning, with neuromorphic vision sensors the first one dating from 1999 [52]. In [53] a neuromorphic cortical-layer microchip for spike-based processing vision systems is presented, which is also an event-based system. Another implementation of an event-based microchip, one that computes 2-D convolutions of video information represented in AER format in real time, can be found in [54]. Some recent applications of these principles can be found in [55,56].

A SCD algorithm can be programmed in two ways. If accurate results are needed as soon as possible, typically the algorithm starts with the classical version method for computing a first version of intermediate images for all the stages. After that, each intermediate image will be updated only when its data source, coming from the precedent stage, has been triggered. These initialization stages have the same temporal cost as the original algorithm, but one must take into account that the temporal cost per image will decrease asymptotically with the SCD algorithm cost when the system is functioning normally. Also, these initialization stages increase the SCD programming code, but this drawback can be considered unimportant taking into account the achieved speed-up. The other SCD programming method avoids these initialization stages. The central part of the algorithm is SCD and the initial classical code for computing a first version of intermediate images for all the stages is not present. Initially the algorithm will not give correct results, but as soon as all the pixels have changed, and all the intermediate pixels have been updated, the algorithm will start to give proper results.

Let us explore how to transform classical image processing operators to the SCD formalism.

### 3.3. Linear Spatial Filtering

As an example of how to implement spatial operators in SCD systems, let us analyze the linear spatial filters or operators. These are very common transformations used for preprocessing or feature extracting. In particular, spatial filtering can be expressed as the systematic application of a convolution mask to all the pixels of the image. Let us say that *G* is the result image of applying the *M* × *M* convolution mask *w*_*i*,*j*_ to the image *I* taken at instant *t*, then each *G*_*x*,*y*_ pixel can be calculated as:

(1) $$G_{x,y}=\sum_{i=\frac{1-M}{2}}^{\frac{M-1}{2}}\sum_{j=\frac{1-M}{2}}^{\frac{M-1}{2}}w_{i,j}I_{x+i,y+j}$$

where *I*_*x*,*y*_ is a pixel of the image *I*. The cost of this transformation, taking into account the multiplication operations, is *nM*^2^, where *n* is the total number of pixels of the image.

Things become different with the SCD sensor. The way of computing the filtered image *G* changes. At the start, an initial full input image *I* for computing is needed, in a classical way, a full filtered image *G*. After that, there will not be a full new image, but a set of *n*′ changing pixels that have been taken at the same time. In this way, the *G* image must be changed only with the contribution of the *n*′ pixels. An individual *I*_*x*,*y*_ pixel taken at instant *t* + 1 contributes to *M* × *M* pixels of *G*, thus this input must be updated, adding the new value and removing the old one. An initial filtered image *G* must be computed in the classical way, but after that, this image is updated using SCD methodology.

**Figure 4.**
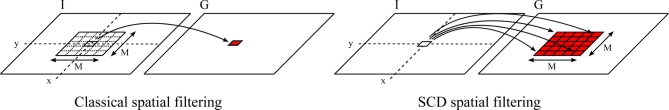
Pixels involved in the computation of linear spatial filtering in the (left) classical and (right) SCD approaches.

Differences between a classic convolution mask (left) and the SCD method (right) are shown in Figure 4. In the classical method, *M*^2^ pixels are needed to compute a single result, and in the SCD method a single pixel change affects to *M*^2^ pixels. It could seem that there are a growing number of operations, but it must be noted that the number of operations remains constant, since for each pixel modification there is a single product. In general, when the SCD camera delivers the pixel *I*_*x*,*y*_ at the instant *t* + 1 the modified pixels in the *G* image are:

(2) $$\begin{array}{cc} G_{x+i,y+j}=G_{x+i,y+j}+w_{-i,-j}\left(I_{x,y}\left(t+1\right)-I_{x,y}\left(t\right)\right) & \\ & \forall i,j\in \left[\frac{1-M}{2},\frac{M-1}{2}\right] \end{array}$$

The difference between the new *I*_*x*,*y*_(*t* + 1) and the old value *I*_*x*,*y*_(*t*) is performed and then *M*^2^ pixels of the *G* image are updated with a single product per pixel. Consequently the cost will be *n*′ *M*_2_, the same as in the classical method but in SCD only the changing pixels are processed. Changing pixels trigger the instructions that depend on them. This is the way SCD processing works. Only the needed operations will be executed and no redundant computations, as happens in a living being, will be performed. In the case of spatial dependency, it is necessary to keep these intermediate images and write the modification algorithms in a data flow manner.

### 3.4. Temporal Dependences

In addition to spatial operators, the extraction of temporal information is important in many image processing algorithms. In particular, it is indispensable for extracting motion from a video sequence. In a simple classical implementation of a first order differentiation, at least two frames taken at different instants are kept in memory, called past and present images. Afterwards, an operator computes results taking into account both images. When a third new complete image is taken, then there is a shift between these images, the past image being replaced by the present image (already in memory) and the present image is replaced by the newly acquired image, this one becoming the new present image. In a classical approach, the temporal operators are applied even if there are no new computations to make, and no matter how many changed pixels are in a new image. This can be seen as a waste of time and resources.

Nevertheless, a SCD based system works in a different manner. There is also an initial image that takes the role of past image, which can be taken with the SCD camera, but now there are no more full-frame incoming images. Afterwards, only a set of changing pixels (acquired at the same time) are read out. The concept of fixed instant can be kept, because these pixels have been taken at the same time, making the transformation from a classical algorithm to the SCD formalism relatively easy. This is a trade-off between a frame-free computation model, as most neuromorphic sensors make, and the full frame image processing classical approach. The SCD policy mimics the non-redundant acquiring, transmitting and processing of the natural world but keeps some concepts of the classical approach, or computing paradigm. To illustrate these ideas let us introduce an example. Let *I*(*t*) be the past image and *I*(*t* + 1) the present image in the video sequence. In a classical approach, a temporal derivative can be approached in the simplest way as the subtraction between both images, this operation being performed for the *n* pixels in the image.

(3) $${\frac{\mathrm{dI}}{\mathrm{dt}}|}_{x,y}\approx \Delta I_{x,y}=I_{x,y}\left(t+1\right)-I_{x,y}\left(t\right)$$

On the other hand, in the SCD approach only the subtraction operation must be performed for the *n*′ read-out pixels, which are the changing pixels. Additionally, only the new pixel *I*_*x*,*y*_(*t* + 1) must update the image *I*(*t*), replacing the old value in the image. If this differentiation is made with intermediate images the process is identical. A changing intermediate pixel triggers the updating of its temporal operator, the intermediate value being updated in the source image. A more complex operator such as a second derivative, or a first derivative mixed with a convolution mask, can be discomposed into simpler spatial and temporal derivatives.

**Figure 5.**
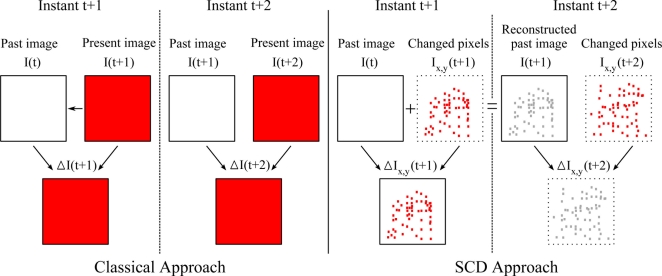
Implementation of the differential operator: Classical **(left)** and SCD **(right)**.

Figure 5 shows the comparison between the differential operation using the classical (left) and the SCD (right) approaches. More complex functions can be converted into the SCD formalism in a similar way. Each new pixel triggers the related operations, taking into account its contribution into the result image of each stage.

## 4. Experimentation

A simple motion detection algorithm is explained in its classical version and in its SCD equivalent description. The motion detection problem has been addressed from many points of view, including bio-inspired architectures and artificial retinas [57,58]. The goal of this experimental implementation is not to solve the motion analysis problem, but to compare the classical implementation of an algorithm to its SCD version, in order to show the benefits in terms of speed-up of our biomimetic approach.

### 4.1. Classical Motion Detection Algorithm

The proposed algorithm computes the mean velocity of a single object in a scene by detecting its edges through a convolution mask, and then calculates its temporal variation subtracting them between two successive frames. If there is a difference (in fact if this difference is greater than a certain threshold depending on the scene) then this pixel is taken into account as belonging to the object. We accept that this is not a very realistic application, but its simplicity will be useful for demonstrating the advantages of the SCD methodology. Some initialization details have been omitted for clarity, since they can be inferred by reading the whole algorithm. The algorithm is as follows:

Compute *EdgeA* (edges of initial image *IA*)

**for** each incoming image *I* **do**

  *X*_0_ ← 0; *Y*_0_ ← 0; *u* ← 0; *v* ← 0;

  **for** each pixel *I*_*x*,*y*_ of the image *IB* **do**

    *EdgeB*_*x*,*y*_ ← 0;

    **for** each pixel *I*_*i*,*j*_ of the surrounding *M* × *M* mask **do**

      *EdgeB*_*x*,*y*_ ← *EdgeB*_*x*,*y*_ + *w*_*i*,*j*_ *I*_*x*+*i*,*y*+*j*_;

    **end for**

    **if** *abs*(*EdgeB*_*x*,*y*_ − *EdgeA*_*x*,*y*_) > *Threshold* **then**

      *diff BA*_*x*,*y*_ ← 1;

      *points* ← *points* + 1;

      *X_center_* ← *X_center_* + *x*;

      *Y_center_* ← *Y_center_* + *y*;

    **else**

      *diff BA*_*x*,*y*_ = ← 0;

    **end if**

  **end for**

  *EdgeA* ← *EdgeB*;

  
$X_{\mathrm{center}}\leftarrow \frac{X_{\mathrm{center}}}{\mathrm{points}}$;

  
$Y_{\mathrm{center}}\leftarrow \frac{Y_{\mathrm{center}}}{\mathrm{points}}$;

  *u* ← *X_center_* − *X*_0_; *v* ← *Y_center_* − *Y*_0_;

  *X*_0_ ← *X_center_*; *Y*_0_ ← *Y_center_*;

**end for**

### 4.2. SCD Motion Detection Algorithm Design

The SCD version of the algorithm described in the previous section is written in a data flow manner, and is prepared to react to each incoming pixel delivered by the SCD camera. Initially it is necessary to compute all the intermediate images through the classical version of the algorithm, so the latency will be the same for obtaining an initial value of the velocity components. The algorithm is as follows:

Compute (*u*, *v*) and all the intermediate values through the classical approach.

*EdgeA* ← *EdgeB*;

**for** each SCD image **do**

  **for** each incoming pixel triplet *x*, *y*, *I*_*x*,*y*_ **do**

    **for** each (*i*, *j*) component of the *M* × *M* mask surrounding *I*_*x*,*y*_ **do**

      *x*′ ← *x* + *i*;

      *y*′ ← *y* + *j*;

      *EdgeB*_*x*′,*y*′_ ← *EdgeB*_*x*′,*y*′_ *w*_−*i*,−*j*_(*I*_*x*,*y*_ − *IB*_*x*,*y*_)

      **if** *abs*(*EdgeB*_*x*′,*y*′_ − *EdgeA*_*x*′,*y*′_) *> Treshold* AND *diff BA*_*x*′,*y*′_ = 0 **then**

        *points* ← *points* + 1;

        
$X_{\mathrm{center}}\leftarrow \frac{(\mathrm{points}-1)X_{\mathrm{center}}+x^{\prime }}{\mathrm{points}}$

        
$Y_{\mathrm{center}}\leftarrow \frac{(\mathrm{points}-1)Y_{\mathrm{center}}+y^{\prime }}{\mathrm{points}}$

      **else if** *abs*(*EdgeB*_*x*′,*y*′_ − *EdgeA*_*x*′,*y*′_) <= *Treshold* AND *diff BA*_*x*′,*y*′_ = 1 **then**

        *points* ← *points* − 1;

        
$X_{\mathrm{center}}\leftarrow \frac{(\mathrm{points}+1)X_{\mathrm{center}}-x^{\prime }}{\mathrm{points}}$

        
$Y_{\mathrm{center}}\leftarrow \frac{(\mathrm{points}+1)Y_{\mathrm{center}}-y^{\prime }}{\mathrm{points}}$

      **end if**

    **end for**

    *IB*_*x*,*y*_ ← *I*_*x*,*y*_

  **end for**

  *u* ← *X_center_* − *X*_0_; *v* ← *Y_center_* − *Y*_0_;

  *X*_0_ ← *X_center_*; *Y*_0_ ← *Y_center_*;

**end for**

In this algorithm, after the initial images, the SCD camera acquires an image at instant *t* and sends the triplet: grey level intensity *I*_*x*,*y*_, together with its coordinates (*x*, *y*), of the changing pixels. In extreme cases, if there were not any moving object, there would not be any grey level change, and thus there would not be any delivered pixel so the system would not process anything, or if all the intensity values had changed, all the pixels would be sent and processed, giving no advantage in terms of reduction of computations. We will show experimentally how, even with a high ratio of changing pixels, a valuable speed-up with the SCD system is achieved.

### 4.3. Experimental Results

Several experiments have been completed, with 32 × 32 pixel simulated images and with the real pixel flow obtained from the SCD camera, in order to point out the advantages of the proposed methodology. First, experiments were performed with conventional images, initially with standard resolution and afterwards down-sampled to 32 × 32 pixels. The idea was to validate algorithm accuracy and speed-up of a classical system with conventional images versus the SCD system with pixel data flow. It is easy to obtain, from a sequence of frames, the equivalent SCD pixel flow. Therefore, it is possible to test the algorithm accuracy speed-up by applying the classical algorithm to the sequence of images and the SCD algorithm to the simulated SCD pixel data flow.

**Figure 6.**
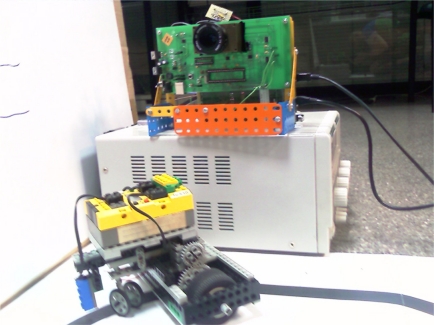
SCD camera and experimental setup.

The classical algorithm was tested initially with 32 × 32 full-frame images to obtain the velocity components of a small moving car. After that, the SCD camera-pixel flow was simulated offline from these still images. The SCD system gave exactly the same velocity component result as the conventional system when all the changing pixels were processed. Thus, the accuracy of SCD processing is proved to be as good as the classical system. If all the pixels are processed, the results are the same as those obtained with the conventional system. Moreover, the achieved speed-up was greater than a factor of 5 with sequences of 20 images, with an average ratio of nearly 50% of changing pixels. Figure 6 shows the SCD camera and the experimental setup for testing the SCD algorithm accuracy.

**Figure 7.**
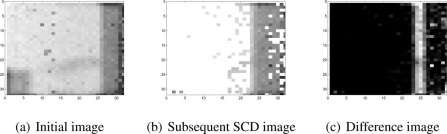
Images from the sensor.

Next, the SCD system was checked with the real pixel flow obtained from the SCD camera. Initially the software was prepared to obtain initial full images from the SCD camera accumulating changes. These images are needed for the initialization part, and after the normal image acquiring and delivering of changing pixels was started. Figure 7 shows an initial image and a subsequent SCD image, together with the difference image. In these images there is a ratio of changing pixels close to 50%, most of them due to the moving object sited in the right-hand part of the image. Other variations appear due to some random noise that can be appreciated in the central parts of the SCD image. These variations are slight variations of the original grey scale, giving a difference compared with the original image near to zero (black colour).

**Figure 8.**
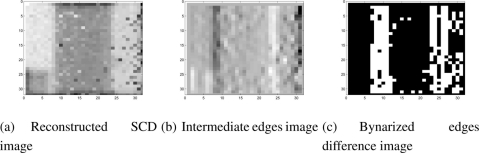
Reconstructed images from the movement detection experiment.

Figure 8 shows several images from the tracking experiment, applying the SCD algorithm to a real pixel-flow acquired with the SCD camera. In this experiment, a vertical strip was placed over a small object moving horizontally from right to left. The scene may appear simple, but with a 32 × 32 resolution sensor, complex objects are not recognizable and simpler geometric forms are better for testing purposes.

The input image shown in Figure 8(a) is the accumulative image that has been reconstructed by adding the changing pixels delivered by the SCD camera. The image is somewhat noisy, since strategies for correcting the image quality in CMOS imagers (fixed pattern noise, *etc.*) have not been applied to show the real sensor output.

**Figure 9.**
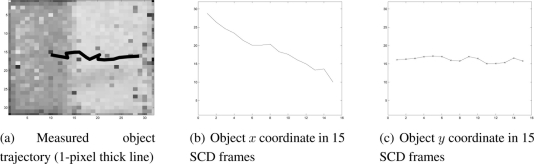
Detected object movement.

In Figure 8(b) the intermediate edges image is shown in the SCD algorithm. To implement the filter stage, a simple Sobel filter for the x coordinate transformed to the SCD formalism has been applied as shown in Section 3.3. The image shows how the edges are correctly detected as well as some spurious small edges corresponding to noisy pixels. Additionally, Figure 8(c) shows the binarized edge difference image from the last stages of the SCD algorithm. From successive difference images, the velocity components (*u*, *v*) are obtained as has been described. With real data flow there is some accuracy loss in the velocity and position data due to the presence of the noisy pixels that are contributing to the position of the object. Figure 9(a) shows the center of mass trajectory with a line 1-pixel thick. Figure 9(a,b) show the center of mass *x* and *y* computed coordinates. Results obtained correspond to what is expected. The *x* coordinate position decreases linearly and the *y* coordinate remains approximately constant due to the fact that the object is only moving from right to left. It must be noted that the lines are not perfectly straight lines due to the 1-pixel maximum accuracy and the contribution of some noisy pixels. Nevertheless, the reasons for this loss of accuracy, due to the use of real images, can be overcome with some correcting techniques.

**Figure 10.**
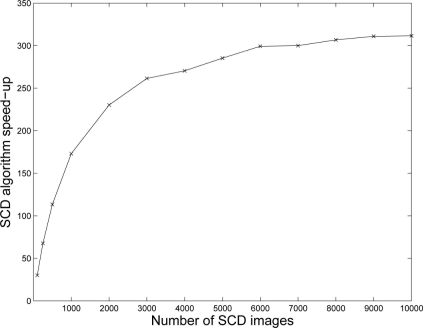
Speed-up execution time of the SCD algorithm versus the original implementation.

At this point, with the SCD sensor we have a more complex algorithm development and lower image quality (at this time) compared to traditional imagers. The motivation for this research project is supported by the speed-up results. Figure 10 shows the speed-up obtained with the moving edge sequence. It must again be noted that these images are the changing pixels obtained and acquired at a certain instant *t* and transmitted, together with their *x* and *y* coordinates, in the order of the magnitude of their change. It is clear that the speed-up will depend on the number of changing pixels. With fewer changing pixels, less redundant operations will be performed by the SCD algorithm compared to the classical version and the algorithm speed-up will increase. In the moving edge sequence, an average of roughly 50% of pixels change. Therefore, we can see that we do not need a low quantity of changing pixels to obtain a valuable speed-up with this strategy. Several experiments with this algorithm have been performed with different sequences giving similar results.

The speed-up shown in Figure 10 increases with the number of images. This result can be explained by taking into account the temporal cost of each version of the algorithm. In the classical versions, there is a fixed initialization cost in the order of processing 3 frames. After that, the cost increases linearly with the number of frames. This happens whether or not there are any changing pixels, and thus the related computations are needed.

In the case of the SCD algorithm, the initialization cost includes: the computation of an initial version of the result, all the intermediate images, plus the cost of the first 3 frames which are processed with the classical algorithm. After that, the temporal cost increases much more slowly since only the changing pixels are processed, resulting in the speed-up increasing with the number of frames. The speed-up asymptotically will approach the quotient between the cost of the central parts of both the original and the SCD algorithms (that is, without the initialization parts). This quotient has been computed, giving a theoretical speed-up slightly over 300, supposing an average number of changing pixels near to 50%. Therefore, Figure 10 shows an asymptote that, as the number of images increases, its speed-up also increases towards the ideal value of more than 300. Of course, this ideal speed-up will vary with the number of changing pixels.

If a very fast moving object that shows very high contrast to the rest of the scene is permanently moving in front of the sensor, then, it is very likely that only the pixels corresponding to the changes produced around this object are read out. In this way, small changes in other parts of the scene cannot be handled at this moment, but they could be processed later if the affected pixel does not change before it is read out. Nevertheless, even under this scenario, depending on the size of the border of the moving object and the rate of the small changes in the background, the throughput of the sensor could be enough to handle the situation. Only very fast changes in a pixel between readouts can be lost if the sensor throughput is overcome.

## 5. Conclusions

A change-driven processing system, instead of a full frame processing system, has been presented. The recently developed 32 × 32 CMOS sensor delivers the changing pixels from the present frame to the last sent frame at each integration time. Several theoretical studies following this strategy were presented in the past with simulated images. The current work presents a working system with real images constructed from the original SCD data flow.

The change-driven processing strategy presented in this paper follows the principles of data flow processing, where a pixel fires the instructions that depend on it, instead of following the imperative programming model. In order to do this, a generic image processing algorithm is implemented as a pipeline which stores intermediate images. A change in an input image pixel triggers the related instructions that change the intermediate values. These changes, at the same time, trigger the instructions that depend on these intermediate changing values.

The methodology and the algorithm implemented are simple, but they show the potential of the method when the motion detection algorithms involved are not very complex. Further experiments with correlation algorithms are being performed at this time.

This way of programming needs an initialization stage that computes an initial version of all the intermediate images and values that must be kept in memory if correct values are desired as soon as possible. The initialization stage is identical to the classical version of the algorithm, producing a larger code as a minor drawback. Moreover, a SCD algorithm needs to keep more data in memory than its classical equivalent. This requirement of extra storage space is not very demanding at present. Other drawbacks, such as the complexity of the system, or even, at this time, the not very high quality of the SCD images, are compensated for by the very high speed-up achieved. The initialization phase can be avoided. In this case, the system will work properly when all the pixels of the intermediate stages have changed.

This kind of system is oriented to real-time image processing, or systems that need very high speed processing. The SCD strategy only delivers new information. There is no redundant data, and the data flow processing system will perform, precisely, the needed computations, with the lowest latency possible. There is a sampling, or snapshot in the classical meaning, each 500 *μ*s instead of the 20 ms of a normal camera. Moreover, there are processing results as soon as the related operations to the changing data are finished.

The hardware needed for implementing a complete SCD-based system is very simple and cheap. The present SCD camera has been implemented with a 32-bit 80 MHz PIC microcontroller just powered by the USB cable, presenting a simple digital interface. The microcontroller can perform the initial steps of the algorithm. The results can be sent through the USB 2.0 link that the microcontroller includes, to higher algorithm layers implemented in an external computer, or additional hardware. This external computing element will follow the principles of the data flow or event-based model. If it is programming code executed by a processor, it will follow the data flow programming adapted to the SCD formalism as stated in this paper.

Further sensors with greater resolution will provide the potential for the implementation of much more complex and accurate algorithms that will operate at the highest possible speed. With greater resolutions, the WTA circuit could be a bottleneck if there were scenes with a lot of changes. In this case, the WTA module could be changed. Future implementations would require further research on the WTA sub-circuit. On the other hand, a possible software bottleneck due to many changes in a greater resolution sensor can be overcome with the implementation of custom data flow architecture in an external FPGA board.
